# Compensation and hazard pay for key workers during an epidemic: an argument from analogy

**DOI:** 10.1136/medethics-2020-106389

**Published:** 2020-05-28

**Authors:** Doug McConnell, Dominic Wilkinson

**Affiliations:** 1 Oxford Uehiro Centre for Practical Ethics, University of Oxford, Oxford, UK; 2 John Radcliffe Hospital, Oxford, UK; 3 Murdoch Children's Research Institute, Melbourne, Victoria, Australia

**Keywords:** public health ethics, health personnel, applied and professional ethics, ethics, health workforce

## Abstract

The COVID-19 pandemic has created unusually challenging and dangerous workplace conditions for key workers. This has prompted calls for key workers to receive a variety of special benefits over and above their normal pay. Here, we consider whether two such benefits are justified: a no-fault compensation scheme for harm caused by an epidemic and hazard pay for the risks and burdens of working during an epidemic. Both forms of benefit are often made available to members of the armed forces for the harms, risks and burdens that come with military service. We argue from analogy that these benefits also ought to be provided to key workers during an epidemic because, like the military, key workers face unavoidable harms, risks and burdens in providing essential public good. The amount of compensation should be proportional to the harm suffered and the amount of hazard pay should be proportional to the risk and burden endured. Therefore, key workers should receive the same amount of compensation and hazard pay as the military where the harms, risks and burdens are equivalent. In the UK, a form of no-fault compensation has recently been made available to the surviving families of key workers who suffer fatal COVID-19 infections. According to our argument, however, it is insufficient because it offers less to key workers than is made available to the families of armed services personnel killed on duty.

## Introduction

During an epidemic, the general public can be protected by social distancing measures, but key workers, such as doctors, nurses, social care workers, first responders, hospital porters and cleaners, supermarket workers, bus drivers and prison wardens, remain at a higher risk of contracting the disease causing the epidemic.^1^ ‘Frontline’ workers face the greatest risk since they are exposed to the virus more frequently and this risk is exacerbated in the absence of adequate personal protective equipment (PPE). In Italy during the COVID-19 pandemic, for example, ~20% of frontline healthcare workers have been infected^2^ while (so far) only ~10% of the Italian general public have been infected.^3^ In the UK, at the time of writing, there have been 108 reported deaths of health and social care workers from COVID-19 over a 3-month period and the final number is expected to be much higher.^4^ In a workforce of ~2.5 million,^5^ this is a fatality rate of 173/million/year which compares with (and adds to) a baseline death rate for health workers of 17–57/million/year.^6^ In a less infectious but more deadly epidemic, Ebola killed ~0.1% of Liberia and Sierra Leone’s general population but ~8% of the countries’ healthcare workers in West Africa (2014–2016).^7^

Key workers will tend to suffer harm from epidemic diseases at a higher rate than the general public, and they will also be under more stress given that risk of harm and from working long shifts in sometimes harrowing conditions. In a survey of UK healthcare workers during the COVID-19 pandemic, 50% reported that their mental health had deteriorated.^8^ Key workers also have to take on a heavier burden than the general public to prevent the transmission of infection to their families.^1^

The issue we will address here is whether key workers are owed special benefits over and above their normal pay for the harms, risks and burdens associated with working during an epidemic. The two forms of special benefits that we will consider have been suggested in the UK in the context of the COVID-19 pandemic, but they would generalise to other epidemics and countries:

1. A no-fault compensation scheme, modelled on the UK’s Armed Forces Compensation Scheme (AFCS), that pays key workers or their families for harm caused by COVID-19.
2. Hazard pay analogous to what the UK Armed Forces receive while on deployment in dangerous areas.

The method we will use to assess whether these benefits ought to be provided to key workers is to assume that they are justified in the military context and then assess the strength of the analogy between key workers and the military. We argue that, if the military deserve these benefits, then key workers during an epidemic do as well.

### Preliminary issues

Before we begin, there are a couple of issues to raise in order to set them aside. Key workers and military personnel are often underpaid for their work, and this injustice is highlighted when they are asked to work during an epidemic or fight in a war. Some might argue that one reason we should provide generous hazard pay or no-fault compensation during an epidemic (or war) is because it would help to rectify this pre-existing injustice. This might be true, but such cases mix the issue of fair base pay with the issue of fair special benefits during epidemics. Because we are interested exclusively in the latter issue here, we will assume that the base pay for key workers and military personnel is fair, at least for a proportion of workers.^1^

We will also assume that the state can afford to pay special benefits during epidemics either through small increases in taxation or cost-savings elsewhere. For wealthy countries facing epidemics similar to COVID-19, this should not be too challenging. Clearly, for poorer countries or countries enduring more harmful epidemics, paying these extra benefits might become too costly. Discussion of what opportunity costs are acceptable in order to pay benefits to key workers during an epidemic would take us too far afield. What our argument does entail, however, is that the grounds for paying extra benefits to key workers are logically related to the grounds for paying extra benefits to the military; so if a country cannot afford to pay these extra benefits to key workers, then it cannot justify continuing to pay equivalent value benefits to the military (at least where the harms, risks and burdens faced by key workers and the military are equivalent).

### No-fault compensation schemes

The AFCS compensates military personnel for harm they suffer on duty.^9^ It is a no-fault scheme which means the claimant need not prove the harm was caused by a negligent party. In contrast, compensation claims in tort law require the claimant to prove in court that the harm they are asking compensation for was caused by a negligent party. There is a debate about how widely available no-fault compensation should be^10^ but, even in New Zealand, where no-fault compensation is available to everybody for accidental harm, there is an additional, more generous compensation scheme for the military.^11^ So, what is it about military service that justifies a generous no-fault compensation scheme and do key workers share those features?

One relevant consideration is that the purpose of the military is to provide substantial public good; they protect the public from foreign threats, exert geopolitical power in the public’s interests and aid the public during natural disasters. In fact, one might go further and argue that the military provides an *essential* public good since, without the military, the democratic state would (eventually) be overtaken by an antidemocratic force. Whether one thinks the military provides an essential or substantial public good, there is a good reason to use public money to ensure an effective military. This reason supports paying and equipping military personnel relatively well. It also provides some support for the provision of a no-fault compensation scheme in that this will incentivise recruitment but it does not specifically support such a scheme over any other incentive.

Stronger support for a no-fault compensation scheme in particular derives from the unusually high danger inherent in military work. As a result of military service, a proportion of people will inevitably be killed, wounded, suffer mental illness and so on. Now, the public is under no moral obligation to compensate for the harm caused by just *any* inherently dangerous work, but if that work provides an essential (or even substantial) public good, then it is only fair that the public compensates workers who are harmed attempting to provide them that good.

Are key workers during an epidemic analogous to military personnel in the relevant ways? It seems indisputable that key workers provide substantial public good during (and outside) epidemics. Without the efforts of frontline healthcare workers during the COVID-19 pandemic, for example, thousands more would die. Those frontline workers can only work because other key workers are providing food, childcare and so on. Indeed, key workers provide an *essential* public good since without food, healthcare, transport, waste disposal et cetera, social order would collapse (hence the term ‘key worker’).^2^ In usual circumstances, the work of key workers involves little danger. However, as outlined above, this changes in the context of an epidemic. Key workers are at greater risk of being infected and harmed by the epidemic than the general public they are working for. So the analogy holds; fairness requires the public to compensate those workers and their families because those workers are inevitably harmed while providing an essential public good.

One might object that a no-fault compensation scheme for key workers is excessive because epidemic conditions are much rarer than instances of military personnel being required to work in dangerous conditions. But the rarity of a situation hardly counts as an excuse to treat people in that situation unfairly and it is not uncommon for no-fault compensation schemes to address transitory causes of harm, for example, cancer caused by exposure to asbestos. Furthermore, if key workers knew in advance that they were covered by a no-fault compensation scheme, they might be more willing to work during an epidemic. This would mitigate the absenteeism which contributes to healthcare systems being overwhelmed.^1 12^

There is a disanalogy between key workers in an epidemic and the military which might appear to undermine the claim of key workers to no-fault compensation. In the armed forces, compensation will only be paid for harm caused through doing one’s job; compensation is typically not paid for injuries suffered while off duty, for example, in a car crash while on leave. It is usually clear if a member of the armed services was harmed while on duty or off duty, but when a key worker dies from an infection, it may be less clear whether the disease was contracted as a result of service or while off duty. Indeed, it will usually be impossible to know where each individual case was contracted.

One can concede that we would not compensate key workers who we knew had contracted the disease outside of work, but argue that we should nevertheless compensate all key workers. This is because we can be confident that the majority of key workers will have contracted the disease at work since social distancing measures reduce transmission in the community. Indeed, even if only a minority of infections were contracted at work, it would be arguably more unjust to leave this minority uncompensated than to compensate a majority who do not deserve it.

If key workers deserve a no-fault compensation scheme analogous to that offered to the military, should they be offered the same amount of compensation as the military? Compensation aims to re-establish the state of affairs prior to the harm suffered; therefore, military personnel and key workers should be compensated equally for equal harm. Military personnel overall might suffer greater harm than key workers, but compensation is paid to individuals not to groups. With this in mind, there is a striking contrast between the UK’s recent announcement of £60 000 to be paid to surviving families of key workers who have died from COVID-19 and the much larger award of four times annual salary plus a lifelong percentage (~60%) of missed earnings paid to surviving families of military personnel who die in service.

Although our argument does not depend on how military personnel are harmed or the kind of work military service entails, it is worth noting that the AFCS explicitly states that it compensates for harm caused by infectious diseases “where service has increased the risk of an individual becoming infected”.^9^ Indeed, this might become relevant because the UK Armed Forces have been brought in to operate mobile COVID-19 testing stations.^13^ This means that a key worker and a soldier could both die of COVID-19 doing very similar work involving very similar risks, but the surviving families in each case would receive substantially different compensation.

If compensation is inadequate, some families of key workers may pursue legal action once the epidemic is over. To win such cases, they will have to show that the harm was caused by negligence, for example, a negligent undersupply of PPE. However, that will provide no relief to the families of those health workers whose deaths were not due to negligence. The personal, emotional and financial loss of those families is no less, but they would be unable to realise any more compensation through the courts.

A lack of PPE will increase the harm suffered in both armed conflicts and epidemics but, even if the lack of PPE was due to negligence, the amount paid in compensation should be proportional to the harm. This is a feature of no-fault schemes; they expedite fair compensation without victims having to prove negligence and dissociate payment for compensation from financial punishment. Potentially negligent parties might still be the target of legal action, but that action would be independent of the compensation process.

### Hazard pay

Since 1970, the UK Armed Forces receive what is called the ‘X Factor’ which increases their base pay by 14.5%. It is “intended to recognise the special conditions of military life, as compared with civilian employment”.^14^ Those special conditions involve things such as the turbulence of relocating between different bases (and the difficulty that creates for one’s spouse to find employment and for children moving between schools), increased risk of injury and mental illness, frequent short-term separation from family, working within a strict command structure where one can be imprisoned for disobeying orders and so on. It also takes into account positive features of military life including the potential for early promotion, access to training and greater job security.

In *addition* to the X Factor, there are a several forms of hazard pay that are paid as per diems when certain conditions are met. Here, we consider two that pay for working in conditions analogous to what key workers face in an epidemic:

The Operational Allowance (OA), currently £29/day or £5281 (tax free) for a 6-month deployment, is “to recognise the significantly increased and enduring nature of the danger in specified operational locations (SOL), over and above that compensated for within the X Factor”. This was paid during the Iraq War, for example.^15^

The Unpleasant Work Allowance (UWA) is paid for working “in conditions involving an exceptional degree of discomfort or fatigue” or exposure “to noxious substance” or for “undertaking other activities that are of an objectionable, or harrowing nature”. The UWA has three levels: Level 1, £2.81/day, for working in confined spaces or with hazardous and noxious substances or in extreme temperatures or a variety of situations where PPE needs to be worn; Level 2, £6.83/day, for working over 4 hours in conditions that qualify for Level 1 but also for the recovery or handling of a small number of un-coffined corpses; Level 3, £20.21/day, for the recovery and exhumation of large numbers of human remains, for example, following a major civil disaster, during humanitarian relief operations or significant loss of military personnel.^15^

The distinction between the X Factor and the forms of hazard pay is instructive. The X Factor pays for risks and burdens that are so commonly part of military service it makes sense to build it into everyone’s pay. The hazard pay per diems recognise that the risks and burdens are sometimes much higher than can be remunerated by the X Factor but, because those situations are relatively rare and some individuals may never face them at all, it makes sense to only pay when they obtain.

The justification for using public money to pay the X Factor and these per diems is similar for the justification to pay no-fault compensation. The public should be prepared to pay the military for taking on risks and burdens where those risks and burdens are necessary to realise substantial or essential public good.

Is there an analogy with key workers during an epidemic such that they too deserve forms of hazard pay? It is difficult to make the case for an X Factor payment to key workers in general because the working conditions created by an epidemic are too rare to be considered an inherent feature of most key worker’s positions. Doctors and nurses who specialise in treating infectious disease might deserve something analogous to an X Factor given their more regular risk of exposure to infectious disease. However, even if an X Factor–style payment were justified in these cases, it would be much smaller than that received by the military because, in ordinary circumstances, the absolute risk of working on specialised infectious disease wards is extremely low, and it does not involve the other components of military life that go towards the X Factor such as working under a strict command structure, the requirement to move locations and so on.

A stronger case can be made for key workers to receive per diem hazard pay, however. During an epidemic, key workers face unavoidable risks and burdens while providing essential public good, and those risks and burdens are well above what most are paid for in their base pay. Supermarket workers, bus drivers and hospital porters, for example, are being paid near minimum wage, some on zero hours contracts, so they cannot plausibly be thought to be sufficiently remunerated for the risks involved in working during an epidemic. Even nurses and physicians, who have a duty to treat,^1^ are working in much rarer circumstances than members of the armed forces when deployed overseas. After all, the X Factor and hazard pay are explicitly outlined in the military’s employment contracts, but nothing like this features in the employment contracts of healthcare workers. So most key workers and even physicians have a case for claiming that the base remuneration and benefits in their contracts fail to fairly compensate them for working in epidemic conditions.

How much hazard pay is due to key workers? The magnitude of hazard pay depends on the risk and burden inherent in one’s work relative to the risk or burden already remunerated by base pay. Given the absence of an X Factor equivalent in most key workers' pay, key workers should be paid more hazard pay than military personnel for the same risk/burden.

But are key workers facing anything like the magnitude of risks and burdens faced by the military? During the period of major combat in the Iraq War, the UK Armed Forces suffered fatalities at a rate of 6 per 1000 personnel years, and this later dropped to below 2 per 1000 personnel years.^16^ For the US military across the entire Iraq War, the fatality rate was about 4 per 1000 personnel years, but in the Vietnam War, it was 22 per 1000 personnel years. Of course, there are also many more seriously wounded in these wars.^17^

If the fatality rate of healthcare workers who contract COVID-19 is about 0.6%^2^ and 20% of frontline key workers become infected,^18^ then ~0.12% (ie, 0.6% of 20%) or ~1/1000 would be expected to die of COVID-19. Since that is over a 3-month period, it is the equivalent of 4–5 fatalities per 1000 personnel years.^3^ But, obviously, more deadly diseases in conditions with less developed healthcare systems can be much more dangerous. The fatality rate of healthcare workers working during the 2014–2016 Ebola epidemic in West Africa was ~50/1000 personnel years in Liberia and Sierra Leone.^7^ So in terms of fatalities, the risks of being involved in armed conflict could be comparable with the risks of working during an epidemic. During recent epidemics, those who survive tend to make a full recovery, so the rate of serious non-fatal injuries is low relative to armed conflict where explosives and small arms fire cause many serious injuries. That said, there is no reason why a future epidemic would not cause serious injury.

Risk of harm is not the only aspect of working during an epidemic that would justify hazard pay. One might have to wear PPE for long periods of time, maintain fastidious sanitation standards well beyond what is usually required for one’s work, take extensive measures to avoid infecting one’s family, see many deaths and distressed families of the dead, move many more cadavers than usual and so on. These unpleasant working conditions can go on for weeks or months at a time. For COVID-19, the conditions seem to fall somewhere between Level 2 and Level 3 of the UWA. When that is combined with the risk of death, key workers in the UK might deserve a hazard pay per diem of ~£30–40 during the worst phase of the COVID-19 pandemic.

## Conclusions

The full scale of the consequences of the COVID-19 pandemic will only become apparent with time. Some have questioned its overall significance, comparing the numbers of patients affected with other endemic and epidemic conditions (such as influenza, cholera or tuberculosis). Whether such comparisons are fair will only be clear when the full scale of the pandemic and its consequences are known. However, one striking feature that clearly distinguishes coronavirus from any of those infectious diseases is the large numbers of key workers who have been seriously affected. In this article, we have argued that the extraordinary impact of the pandemic on healthcare and other key workers requires an extraordinary response. We have drawn the analogy with no-fault compensation and hazard pay offered to military personnel. If such measures are justified in the case of the military, they are also justified in the case of key workers during a severe epidemic where they are subject to substantial personal risk and unusually burdensome workplace conditions.

## Data Availability

The are no data in this work.

## Footnotes

If (as we argue) it is justified to provide additional compensation in the setting of fair base pay, that conclusion will necessarily translate to (and may be strengthened) in cases where workers receive unfair base pay.

It seems likely that key workers have provided the UK more benefit during the COVID-19 pandemic, for example, than the Armed Forces have provided in their 21st Century overseas deployments.

The UK rate appears significantly lower than this at the moment (see above), but it is not clear whether that is due to a lower infection rate, a lower case fatality rate or a delay in confirming deaths.
